# Post-mortem 11.7 Tesla Magnetic Resonance Imaging vs. Polarized Light Imaging Microscopy to Measure the Angle and Orientation of Dorsal Root Afferents in the Human Cervical Dorsal Root Entry Zone

**DOI:** 10.3389/fnana.2019.00066

**Published:** 2019-07-02

**Authors:** Dylan Jozef Hendrik Augustinus Henssen, Rosanna Christina Weber, Jesse de Boef, Jeroen Mollink, Tamas Kozicz, Erkan Kurt, Anne-Marie van Cappellen van Walsum

**Affiliations:** ^1^Department of Anatomy, Donders Institute for Brain, Cognition & Behavior, Radboud University Medical Center, Nijmegen, Netherlands; ^2^Unit of Functional Neurosurgery, Department of Neurosurgery, Radboud University Medical Center, Nijmegen, Netherlands; ^3^Nuffield Department of Clinical Neurosciences, Wellcome Centre for Integrative Neuroimaging, Oxford Centre for Functional MRI of the Brain (FMRIB), University of Oxford, Oxford, United Kingdom; ^4^Department of Clinical Genomics, Mayo Clinic Minnesota, Rochester, MN, United States

**Keywords:** anatomy, brachial plexus avulsion injury, dorsal root entry zone lesioning, dorsal root entry zone, magnetic resonance imaging, polarized light imaging

## Abstract

**Background**: Destruction of the afferents by dorsal root entry zone (DREZ) surgery may be an effective treatment of intractable neuropathic pain, though it remains a high-risk surgical intervention. Potential complications due to the lesioning of structures within the cervical spinal cord other than the DREZ can be minimized by accurate knowledge of the optimal insertion angle [i.e., the angle between the DREZ and the posterior median sulcus (PMS)]. The employed insertion angle was based on measurements between the DREZ and the PMS on post-mortem transverse slices. However, new, more sophisticated imaging techniques are currently available and are thought to yield higher spatial resolution and more accurate images.

**Obejctive**: This article measures the angle between the DREZ and the PMS on 11.7T post-mortem magnetic resonance images and compares these findings with polarized light imaging (PLI) microscopy images of the same specimens in order to quantify fiber orientation within the DREZ.

**Methods**: To visualize the anatomy of the cervical DREZ, magnetic resonance imaging (MRI), diffusion-weighted MRI (dMRI), probabilistic tractography, and PLI were performed on three post-mortem human cervical spinal cords at level C5–C6. The MR data was used to measure the angle between the DREZ and the PMS. MR images were complemented by probabilistic tractography results. Then, the orientation of fibers within the DREZ was quantified by use of PLI microscopy.

**Results**: Median angle between the DREZ and the PMS, as measured on MR-images, was found to be 40.1° (ranging from 34.2° to 49.1°) and 39.8° (ranging from 31.1° to 47.8°) in the left and right hemicord, respectively. Median fiber orientation within the DREZ, as quantified by PLI, was 28.5° (ranging from 12.0° to 44.3°) and 27.7° (ranging from 8.5° to 38.1°) in the left and right hemicord, respectively.

**Conclusion**: Our study, which provides an improved understanding of the anatomy of the DREZ, the angle between the DREZ and the PMS and the median fiber orientation within the DREZ, could contribute to safer DREZ-lesioning surgery to treat chronic neuropathic pain in the future.

## Introduction

The gate control theory (Melzack and Wall, 1965) is based on anatomical and physiological observations that non-nociceptive stimuli are transmitted *via* three separate systems within the spinal cord: (1) the substantia gelatinosa; (2) relay cells in the dorsal horn; and (3) the ascending fibers within the dorsal column (Mendell and Wall, 1964; Melzack and Wall, 1965). The gate control theory proposed that non-nociceptive input closes a “gate” to nociceptive input, preventing these noxious signals from traveling to the central regions where these signals are processed as pain. These nerve gates were hypothesized to lie within the dorsal horn of the spinal cord, drawing the attention of neurosurgeons to this region as a modulator for pain (Sindou and Jeanmonod, 1989). This inspired Sindou et al. (1974) to selectively destroy the fibers that run in the dorsal root entry zone (DREZ).

The term DREZ has been used for many years with disparate meanings. In this article, the term refers to the plane of insertion of the dorsal root fibers into the spinal cord, and includes the terminal part of the dorsal roots, Lissauer’s tract and the superficial laminae of the dorsal spinal horn. For neurosurgeons, a new therapy called DREZ lesioning was further developed by Nashold in 1979 (Nashold and Ostdahl, 1979) in order to interrupt the sensory pathways completely by the use of microsurgical coagulations, radiofrequency thermocoagulation, laser-induced lesioning or ultrasound-induced lesioning (Burchiel and Sindou, 2011). Over the years, DREZ-lesioning has been reported to be an effective treatment of post-avulsion pain (Friedman et al., 1984; Friedman and Bullitt, 1988; Sindou et al., 2005; Chen and Tu, 2006; Aichaoui et al., 2011). Unfortunately, despite extensive preoperative magnetic resonance imaging (MRI) and intraoperative neurophysiological monitoring, DREZ lesioning remains a high-risk surgical intervention with potential adverse events, including both over-lesioning and under-lesioning complications (Spaić et al., 2002; Chen and Tu, 2006; Xiang et al., 2008; Chun et al., 2011; Haninec et al., 2014). These complications may in part result from uncertainty regarding the insertion angle of the DREZ-lesioning device. This neurosurgical disagreement reflects an anatomical controversy over, on the one hand, the angle between the DREZ and the posterior median sulcus (PMS) and the median fiber orientation within the DREZ the fiber orientation of afferents within the DREZ, on the other hand.

According to initial reports, DREZ lesioning should be performed at an angle of 25° (Nashold and Ostdahl, 1979; Rawlings et al., 1989); subsequently, this angle was adjusted to 40° due to the study of Xiang et al. (2008). However, in the study of Xiang et al. (2008) the insertion angle was calculated by linear measurements between two points (e.g., the angle between the PMS and the dorsal roots) in non-pathological, post-mortem specimens. New state-of-the-art techniques could contribute to the ongoing discussion regarding the orientation of the primary afferents in the DREZ. For example, 11.7T post-mortem MRI has been used successfully to obtain high-quality structural images and to map fiber architecture. In addition, polarized light imaging (PLI) microscopy has been reported as a well-equipped method to validate post-mortem MR findings as it is regarded as a gold standard for investigating myeloarchitecture, fiber orientation in particular (Axer et al., 2011a; Axer H. et al., 2011). This study defines the anatomy of the human cervical spinal cord by use of these innovative imaging techniques and aims to measure the angle between the DREZ and the PMS on 11.7T post-mortem magnetic resonance images and to compare these findings with PLI microscopy images of the same specimens in order to quantify fiber orientation within the DREZ.

## Materials and Methods

### Ethical Statement

Specimens were acquired *via* the body donor program at the Department of Anatomy at our center. All body donors in this program signed a written informed consent during lifetime permitting the use of their body and parts for science and teaching. The body donor program of the Radboud University Medical Center was approved by the national medical ethical committee of Netherlands and legislated under Dutch law. This study was performed with the approval of the medical ethical committee of the region Arnhem–Nijmegen.

### Acquisition of the Specimens

Three post-mortem spinal cords were obtained from our center’s body donor program. The three donors had no known neurological diseases. All specimens were perfused *via* the femoral artery using 10% formalin (~38% solution of formaldehyde gas in tap water) within 24 h after death. Specimens were fixed in 7.7% formalin separately for at least 6 months. The three cervical segments were dissected using a posterior laminectomy at level C2–C6. Each specimen was placed in a prone position. An incision was made in the midline from the external occipital protuberance to the vertebra prominens. Next, the connective tissue and superficial fascia covering the muscles were pulled aside using retractors as were the muscles in the neck, to expose the posterior vertebral arches C4 to C7. The segmental level of each spinal nerve was defined and confirmed to the corresponding vertebrae. The posterior arches were removed using a Leksell rongeur, which left the dura and the arachnoid membrane exposed. The dura was opened with a longitudinal incision along the midline at spinal cord level C5 and C6. The dorsal and ventral rootlets C5–C6 were used as a reference point for the spinal cord level. Thereafter, the rootlets were incised, and circumferential cuts into the spinal cord were made superior to the spinal cord C5 level and inferior to the spinal cord C6 level. The spinal cords were then removed and immersed in 7% formalin until further analysis. Table 1 provides an overview of the characteristics of the specimens used in this study.

**Table 1:** Characteristics of the specimens used in this study.

Characteristics	Subject 1	Subject 2	Subject 3
*Age*	72	68	91
*Gender*	Male	Male	Male
*Cause of death*	Esophageal cancer	Pneumonia	Cardiac arrest
*Post-mortem interval*	14 h	7 h	22 h

### Magnetic Resonance Image Acquisition

Prior to scanning, the three spinal cords were stored in a phosphate-buffered saline solution (PBS 0.1 M, pH 7.4) for 7 days as formalin is known to decrease T2 relaxation rate of post-mortem tissues (D’Arceuil and de Crespigny, 2007; Schmierer et al., 2008). Next, tissues were placed in a 100 ml syringe, filled with perfluoropolyether (Fomblin^®^, Solvay Solexis Inc., West Deptford, NJ, USA), a hydrogen-free liquid matching the susceptibility of nervous tissue, 24 h prior to scanning for immersion. Each specimen was imaged at 20° Celsius on an 11.7 T Bruker BioSpec preclinical MR system (Bruker BioSpec 117/16) using a birdcage coil. Scanning parameters are provided in Table 2. The applied MR protocol was adapted from an empirically designed protocol reported in the literature (Kleinnijenhuis et al., 2013) and has been previously reported by our group (Henssen et al., 2019).

**Table 2:** Characteristics of the applied MRI protocol.

**Diffusion Weighted Spin echo planar imaging**	
*T*_E_	30.70 ms
*T*_R_	13.75 ms
*α*	30°
Δ	12.5 ms
*δ*	4.0 ms
*Number of directions*	256 gradient directions
*Number of averages*	2
*Number of q = 10 mm^−1^ (b = 0 equivalent)*	6
*Voxel size*	0.5 × 0.5 × 0.5 mm^3^
*b-value (equivalent)*	~4,000 s/mm^2^
**True Fast Imaging with Steady State Free Precession (TRUFI, anatomical)**	
*T*_E_	7 ms
*T*_R_	3,314 ms
*α*	20°
*Voxel size*	0.25 × 0.25 × 0.25 mm

*T_E_, Echo time, represents the time from the center of the RF-pulse to the center of the echo; T_R_, Repetition time, the length of time between corresponding consecutive points on a repeating series of pulses and echoes; α, Flip angle, amount of rotation the magnetization experiences during application of a radiofrequency (RF) pulse; b-value, measures the degree of diffusion weighting applied; δ, Duration of the pulse; Δ, Time between the two pulses*.

### Probabilistic Tractography

All processing of the MR data was performed using FSL (Jenkinson et al., 2012). To correct for eddy current artifacts and displacement between the different diffusion images, an eddy current correction was applied (Andersson and Sotiropoulos, 2015). The diffusion-weighted MRI (dMRI) data were pre-processed for tractography using the BedpostX algorithm that models multiple fiber orientations (*n* = 3) at each voxel (Behrens et al., 2007). Probabilistic tractography, using the Probtrackx2 algorithm (Behrens et al., 2007), delineated the afferent pathways of the dorsal root in the three specimens. A seed mask was manually placed in the DREZ in the acquired fractional anisotropy (FA) maps. Streamlines were drawn from each seed-voxel (*n* = 50,000 streamlines per voxel). A waypoint mask was manually placed at the level of the anterior white commissure (AWC). Maximum intensity projection (MIP) were produced to optimize visualization of the tractography results (Foxley et al., 2014).

### Sectioning

After scanning, the spinal cords were sectioned to visualize the fiber systems at high resolution with PLI microscopy and with histological staining techniques. Prior to sectioning, tissues were cryoprotected by immersing the samples in 30% sucrose in 0.1 M PBS for 5 days at a temperature of 4°C. The specimens were fast-frozen and sectioned using a HM 450 Sliding Microtome (Thermo Fisher Scientific Inc., Waltham, MA, USA). A thickness of 100 μm was used in a series of five sections, every first and second slice was mounted for myelin staining and PLI microscopy, yielding an interslice distance of 500 μm for each technique. All mounted sections were cover-slipped using the mounting medium polyvinylpyrrolidone.

### Polarized Light Imaging

PLI is based on the optical birefringence of nerve fibers, reflecting the arrangement of lipid layers in the myelin sheaths surrounding axons; PLI can be used to visualize fiber orientation in histological slices. More in-depth description of the physics supporting PLI has been described elsewhere (Axer et al., 2011a,b). A Zeiss ImagerA2 fluorescence microscope (Carl Zeiss Microscopy GmbH, Jena, Germany), upgraded with a stationary polarizer, a quarter-wave plate and a rotating polarizer, was used to acquire high-resolution PLI images of the spinal cords. In the set-up used, light passes through a linear polarizer and a quarter wave plates with its fast-axis oriented at 45° with respect to the polarizer. This creates circularly polarized light that passes through the specimen, followed by another polarizer which captures the change in polarization. Due to the simultaneous rotation of all polarizing filters, the birefringence of the specimen is systematically imaged at discrete equidistant angles from 0° to 180°. Together with a 1.25× magnifying objective, this yielded a spatial resolution of approximately 4 μm/pixel. This polarization microscope set-up was used to scan a grid of high-resolution images. Slices were therefore divided into different field of views (FOVs) to cover the entire specimen. Only the green channel was used for further processing, as the quarter-wave plate was designed for this wavelength. A set of background images was acquired for every rotation angle to correct for inhomogeneous background illumination. Background correction of the images was performed as previously described (Dammers et al., 2010).

Three different parameters could be derived from the raw PLI data by fitting the light intensity at each pixel to a sinusoid: (1) the phase of the sinusoid; (2) the phase shift induced to the light wave; and (3) the average amount of light passing through the tissue. Each of these parameters provided a different PLI map. The phase of the sinusoid provided an in-plane orientation map; the phase shift induced to the light wave provided a retardance map; and the transmittance map was calculated as the average amount of light passing through the tissue. By combining the retardance-map and the in-plane orientation-map, the fiber orientation map was acquired, allowing for which visualization of the direction of myelinated fibers within the tissue (Axer et al., 2011a). All maps of the different FOVs were stitched to provide an overview of the entire slice, using in-house software written in MATLAB© (The MathWorks Inc., 1994–2017).

### Angular Measurements on MR Images and Quantified Fiber Orientation Measurements on PLI Microscopy Images

MR data could be used to perform angular measurements. A total of 15 MR slices were used for each specimen, spanning 15 mm at the level C5–C6. Using trigonometry, the angle between the PMS and the entrance of the dorsal root was measured.

PLI data could be used to measure median fiber orientation. A total of 15 PLI sections were used for each specimen, spanning 15 mm at the level C5–C6. A manually drawn region of interest was placed at the position of the left and right dorsal root in the in-plane maps. The region of interest covered the DREZ and the most dorsal tip of the dorsal horn. Using in-house written software in MATLAB© (*The MathWorks Inc., 1994–2017*), the average in-plane orientation of each region of interest was determined, taking the PMS as reference.

IBM SPSS Statistics version 22 (*IBM Corp. Released 2013. IBM SPSS Statistics for Windows, Version 22.0. Armonk, NY, USA: IBM Corp*.) was used for descriptive statistics and values are represented as mean ± standard deviation.

## Results

### Structural and Diffusion MR Findings

**Figure 1:**
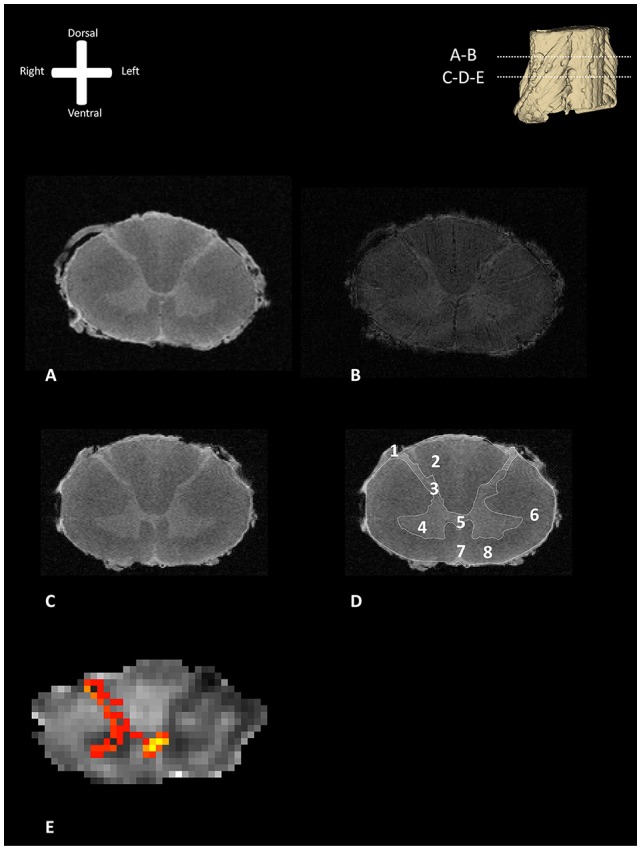
Transverse section of one of the human cervical spinal cords (specimen 1) as depicted with different MR-techniques. 3D model in the viewers right corner shows the level of the transverse sections depicted as subfigures **(A,B)**. **(A)** T1-weighted anatomical image of a transverse section of the human cervical spinal cord. **(B)** T2^*^-weighted anatomical image of a transverse section of the human cervical spinal cord. **(C)** T1-weighted anatomical scan without annotations. **(D)** T1-weighted anatomical scan with annotations; 1: dorsal root entry zone (DREZ), 2: dorsal columns, 3; dorsal horn, 4: ventral horn, 5: posterior gray commissure, 6: lateral column, 7: anterior median fissure, 8: anterior column. **(E)** Maximum intensity projection (MIP) of the tractography reconstruction of a transverse section of the spinal cord showing afferent pathways. Tractography is superimposed on a fractional anisotropy (FA) map. The afferent bundle bifurcates into a lateral and medial bundle. The lateral bundle contributes to the uncrossed spinothalamic tract, whereas the medial bundle decussates *via* the anterior white commissure (AWC) to contribute to the crossed spinothalamic tract.

Figure 1 shows exemplary axial slices of the three different MR protocols. Figure 1A depicts a structural, T1-weighted, 11.7 T transverse section of the human spinal cord at the level of entrance of the dorsal rootlets of C5–C6. Figure 1B shows the T2*-weighted image of the same transverse section, showing the susceptibility artifact which was encountered due to the small amounts of remaining coagulated blood in the spinal cord microvasculature. Figure 1C depicts a more caudal T1-weighted axial slice of the human spinal cord. In Figure 1D, the most important anatomical structures and (internal) landmarks are provided. Figure 1E shows the probabilistic tractography results, projected as MIP results over the FA maps. From the dorsal horn, the probabilistic tractography result shows a more probabilistic tract coursing towards the center of the spinal cord *via* the AWC.

### PLI Parameter Maps

**Figure 2:**
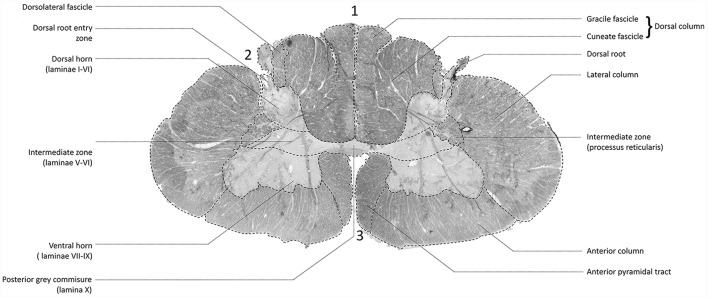
Transmittance image of a transverse section of the cervical spinal cord at level C5–C6 (specimen 2). 1: posterior median sulcus (PMS). 2: dorsolateral sulcus, 3: anterior median fissure.

**Figure 3:**
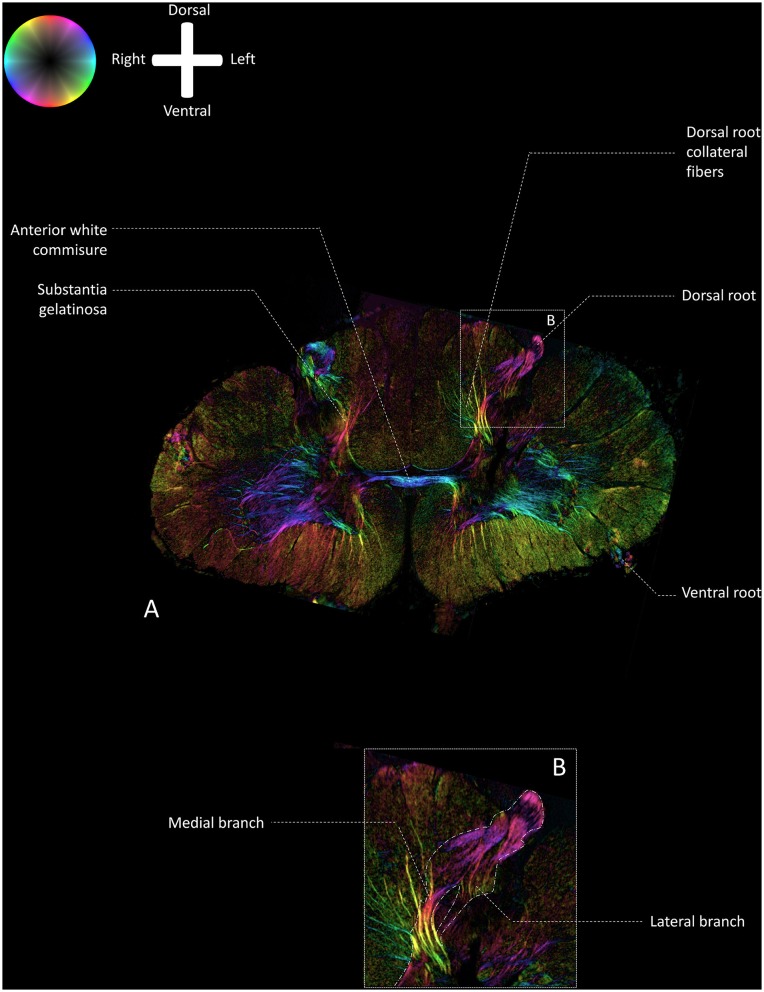
Fiber orientation image of the a transverse section (100 μm) at level C5–C6 of the cervical human spinal cord (specimen 2; similar section as depicted in Figure 2). The fiber orientation is defined by the color sphere and the intensity modulated by the density of in-plane myelinated fibers. **(A)** Overview of the white matter pathways in the cervical spinal cord. **(B)** Enlargement of inset in Panel **A**. The medial and lateral bundles of the entering dorsal root can be recognized. The lateral bundle can be seen as a low-intensity area, whereas the medial bundle can be followed up until the deeper layers in the dorsal horn.

Figure 2 depicts a transmittance image of a transverse section of the human cervical spinal cord. In Figure 3A, the dorsal roots can be observed to enter the spinal cord of the second specimen through the DREZ, shown in the image as purple colored tract. After entering the spinal cord, two tracts can be distinguished: a lateral and medial bundle (Figure 3B). The medial bundle can be observed to spread out and course towards the deeper layers of the dorsal horn. The yellow fibers likely correspond to heavily-myelinated dorsal root fiber collaterals, as these fibers were observed to disperse towards the dorsal columns (Nieuwenhuys et al., 2007; Figure 3A). The lateral bundle (Figure 3B) can be observed as well, although lower in PLI signal intensity. Some of these fibers were observed to course towards the contralateral hemicord *via* the AWC. The medial and lateral bundles were observed in each specimen.

### Angular Measurements on MR Images vs. Quantified Fiber Orientation Measurements on PLI Microscopy Images

**Figure 4:**
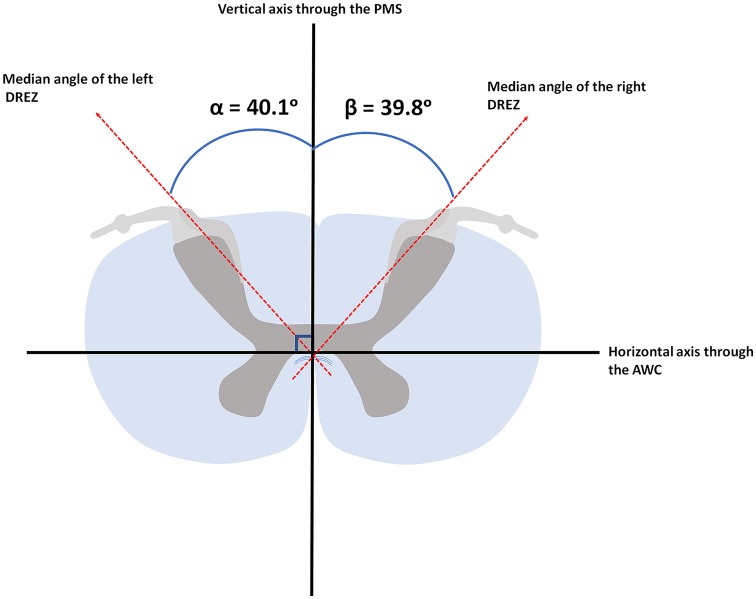
Median angular measurements of the DREZ within the left and right dorsal root. AWC, anterior white commissure; PMS, posterior median sulcus.

Based on the 11.7 Tesla, T1-weighted MR-images, the median angle between the DREZ and the PMS in the left cervical hemicord was found to be 40.1° (ranging from 34.2° to 49.1°), whereas the median angle between the DREZ and the PMS in the right cervical hemicord was 39.8° (ranging from 31.1° to 47.8°; Figure 4).

**Figure 5:**
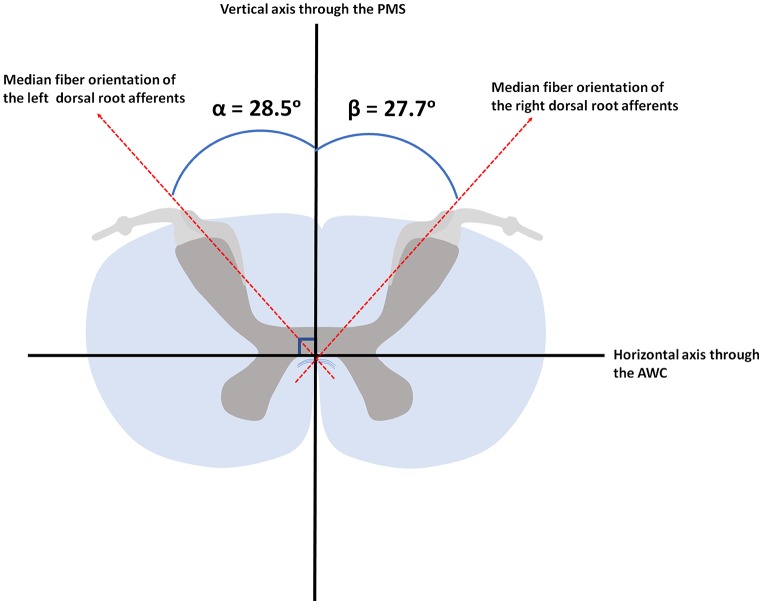
Median orientation of the afferents within the left and right dorsal root. AWC, anterior white commissure; PMS, posterior median sulcus.

The PLI microscopy data quantified fiber orientation within the DREZ. The median orientation of fibers within the DREZ was found to be 28.5° (ranging from 12.0° to 44.3°) with regard to the PMS in the left cervical hemicord. The entering fibers showed to have a median fiber orientation of 27.7° (ranging from 8.5° to 38.1°) with regard to the PMS in the right cervical hemicord (Figure 5).

## Discussion

### Anatomy as Visualized by PLI in the Context of the Existing Anatomical Literature

The present study visualizes the white matter anatomy of the spinal cord and shows the subdivision of the medial and lateral bundles of the primary afferents of the dorsal rootlets. The lateral bundle had a lower PLI signal, indicating that this bundle contains less myelinated fibers, in agreement with early anatomical investigations (Cajal, 1909; Scheibel and Scheibel, 1968) and the study of Sindou et al. (1974). These articles showed that the lateral bundle consisted mainly of small caliber, less myelinated (Aδ and C) fibers, whereas the medial bundle contained large caliber (Aβ fibers; Sindou et al., 1974). This is expected since the PLI signal arises mainly from the birefringent properties of myelin (Axer et al., 2011a). Traditionally, the lateral bundle of the primary afferents has been suggested as the ideal target of selective DREZ-lesioning in intractable pain (Sindou et al., 1974).

PLI data was not capable of providing specific in-depth information that could be derived from animal-based tract-tracing studies. For example, such studies showed that, after synapsing with individual spinal neurons, the primary afferents transmitted information to the ascending systems in both uncrossed and crossed fashion. In 1989, the primate spinothalamic pathways were investigated by injection of 2% wheat germ agglutinin-conjugated horseradish peroxidase in the thalamus. Using this technique, 17% of the retrogradely-labeled spinothalamic tract cells were found to have an ipsilateral projection (Apkarian and Hodge, 1989). The same study showed that a dorsolateral spinothalamic tract exists in Old World monkeys, next to the ventrolateral spinothalamic tract containing the anterior and lateral spinothalamic tract. The dorsolateral spinothalamic tract mainly comprised crossing axons that originate from dorsal horn layer I, whereas the ventral spinothalamic tract comprised crossing axons originating from multiple laminae (Apkarian and Hodge, 1989).

### Clinical Relevance

Clinically, the median fiber orientation measurements of this study offer insights into potential value for future DREZ-lesioning therapy. Post-operative complications of DREZ-lesioning are common, including poor outcomes arising from over-lesioning or under-lesioning (Spaić et al., 2002; Chen and Tu, 2006; Xiang et al., 2008; Chun et al., 2011; Haninec et al., 2014). Adverse event associated with under-lesioning includes ineffective pain relief (Rawlings et al., 1989), whereas over-lesioning can lead to paralysis or dysesthesia of more caudal segments of the human body, due to the destruction of adjacent anatomical pathways in the spinal cord, including the dorsal spinocerebellar, lateral corticospinal and rubrospinal tracts and the dorsal fasciculus (Rawlings et al., 1989). The occurrence of sensory-, motor- and genito-sphincterian deficits as over-lesioning complications depends on a variety of factors (Rawlings et al., 1989; Burchiel and Sindou, 2011), including the operative procedure, the chosen insertion angle, and the chronic pain condition, which corresponds with the level at which DREZ lesioning is carried out (Rawlings et al., 1989; Burchiel and Sindou, 2011). To minimize the risk of complications, the appropriately sized lesion and the correct angle are of vital importance. The first studies on DREZ-lesioning reported an insertion angle of 25° with regard to the PMS (Nashold and Ostdahl, 1979; Rawlings et al., 1989). However, later studies reported insertion angles of up to 40° (Xiang et al., 2008). None of these studies, however, investigated the orientation of fibers as demonstrated here by the PLI data. The aforementioned studies performed linear measurements between two points (e.g., the angle between the PMS and the dorsal roots), which was reproduced here on the 11.7 Tesla structural MR data. The angular measurements of the 11.7 Tesla MR data presented here, closely resembled the angles reported in the literature (Xiang et al., 2008). The median fiber orientation results presented here imply that an insertion angle of approximately 25° should be recommended for neurosurgeons that perform DREZ-lesioning in patients that suffer from neuropathic pain with non-pathological spinal cords (i.e., phantom limb pain).

Regarding the range of the measurements, the authors wish to underline the importance of combining these insights with preoperative clinical neurophysiological monitoring. Certain groups of patients that undergo DREZ-lesioning have deformed spinal cords. One of the most notable examples comprises brachial plexus avulsion (BPA) patients. BPA is a traumatic injury in which the cervical spinal nerves get disconnected from the cervical spinal cord (Midha, 1997; Stewart and Black, 2004). Although the primary cervical afferents are avulsed, the corresponding cell bodies of the neurons in the dorsal horn remain intact (Teixeira et al., 2015). Thereby, the second-order neurons are deafferented and no longer receive physiological input, which can lead these second-order neurons to develop ectopic excitability, tending to fire impulses spontaneously to supraspinal third-order neurons, for example, those that lie within the thalamus and project to parts of the pain matrix within the brain. This process is believed to underlie neuropathic, post-avulsion pain, which occurs in 54.5% of BPA patients (Zhou et al., 2017). In 2005, a study showed that 78% of all brachial dorsal roots were damaged, 79% of which were totally avulsed and 21% of which were partially avulsed or atrophic. Additional impairments of the spinal cord were observed in 49% of the reported patients, including spinal cord atrophy, deviations and distortions of the spinal cord. Furthermore, focal gliosis and microcysts were found inside the dorsal horn in 36% of the patients (Sindou et al., 2005). These deformations complicate the translation of the presented findings directly into future DREZ-lesioning procedures.

### Strengths and Limitations

A strength of this study is the implementation of PLI to re-investigate the anatomy of white matter tracts in the spinal cords. PLI, which can provide detailed insights into the architecture of the nervous system, has mainly been used to visualize white matter tracts in the brains of rodents and humans (Larsen et al., 2007; Axer et al., 2011a). This study showed the superiority of PLI over post-mortem high-field (11.7 T) MRI, especially for visualizing the transversely orientated fibers, reflecting the mechanisms underlying PLI. As the longitudinally oriented major pathways and their myelin sheets run parallel to the direction of the polarized light in the set up used, the birefringence of these pathways is limited, whereas the myelinated fibers that run perpendicular to the polarized light are clearly visualized using PLI. A limitation of this study is that only the transverse anatomical plane was investigated. Future investigations using PLI to study the anatomy of the white matter tracts in the spinal cord should consider imaging the sagittal planes of the spinal cord as well. By adding this anatomical plane to the results, the three-dimensional anatomy of the dorsal horn, as described by Scheibel and Scheibel (1968), could be reproduced in a modern way. Only non-pathological human spinal cords were used in this study. Accordingly, our results will be of limited value when treating patients with deformed spinal cords.

## Conclusion

Our findings regarding the angle of entrance of the cervical dorsal root may contribute to a better understanding of over-lesioning and under-lesioning complications in patients that undergo DREZ-lesioning therapy to treat chronic neuropathic pain.

## Ethics Statement

This study was carried out in accordance with the recommendations of the CMO (Commissie Mensgebonden Onderzoek) region Arnhem-Nijmegen, Netherlands. Specimens were acquired *via* the body donor program at the Department of Anatomy at our center. All body donors in this program signed a written informed consent during lifetime permitting the use of their body and parts for science and teaching.

## Author Contributions

All authors provided input for this article. DH, JB, RW, JM and EK undertook the actions of collecting the materials and acquiring the data. A-MCW, EK and TK provided the team with valuable anatomical and clinical feedback.

## Conflict of Interest Statement

The authors declare that the research was conducted in the absence of any commercial or financial relationships that could be construed as a potential conflict of interest.
